# Early pannus after transcatheter heart valve implantation leading to delayed coronary obstruction: a case report

**DOI:** 10.1093/ehjcr/ytag243

**Published:** 2026-04-13

**Authors:** Louis Perrard, Alexandre Lafont, Mariama Akodad, Patrick Nataf, Emmanuel Gall

**Affiliations:** Université Paris-Cité, Department of Cardiology, University Hospital of Lariboisiere, (Assistance Publique des Hôpitaux de Paris, AP-HP), 2 rue Ambroise Paré, Paris 75010, France; Université Paris-Cité, Inserm MASCOT - UMRS 942, 2 rue Ambroise Paré, Paris 75010, France; MIRACL.ai Laboratory, Multimodality Imaging for Research and Artificial Intelligence Core Laboratory, University Hospital of Lariboisiere (AP-HP), 2 rue Ambroise Paré, Paris 75010, France; Department of Cardiology, University Hospital of Amiens, 1 rue du Professeur Christian Cabrol, Amiens 80000, France; Université Paris-Cité, Department of Cardiology, University Hospital of Lariboisiere, (Assistance Publique des Hôpitaux de Paris, AP-HP), 2 rue Ambroise Paré, Paris 75010, France; Université Paris-Cité, Inserm MASCOT - UMRS 942, 2 rue Ambroise Paré, Paris 75010, France; MIRACL.ai Laboratory, Multimodality Imaging for Research and Artificial Intelligence Core Laboratory, University Hospital of Lariboisiere (AP-HP), 2 rue Ambroise Paré, Paris 75010, France; Institut Cardiovasculaire Paris Sud, Hôpital Privé Jacques Cartier, Ramsay Santé, 6 avenue du Noyer Lambert, Massy 91300, France; Service de Chirurgie Cardiaque, Hôpital Bichat, APHP, 46 rue Henri Huchard, Paris 75010, France; Université Paris-Cité, Department of Cardiology, University Hospital of Lariboisiere, (Assistance Publique des Hôpitaux de Paris, AP-HP), 2 rue Ambroise Paré, Paris 75010, France; Université Paris-Cité, Inserm MASCOT - UMRS 942, 2 rue Ambroise Paré, Paris 75010, France; MIRACL.ai Laboratory, Multimodality Imaging for Research and Artificial Intelligence Core Laboratory, University Hospital of Lariboisiere (AP-HP), 2 rue Ambroise Paré, Paris 75010, France

**Keywords:** Transcatheter heart valve, Multimodality imaging, Computed tomography, Early pannus, Coronary obstruction, Case report

## Abstract

**Background:**

Coronary obstruction after transcatheter aortic valve implantation (TAVI) is a rare complication typically occurring acutely. Delayed coronary obstruction (DCO) due to early pannus formation is exceptionally uncommon.

**Case summary:**

A 74-year-old woman with no coronary artery disease underwent TAVI with a self-expandable 26-mm Evolut Pro + (Medtronic) transcatheter heart valve (THV) six months prior. She presented with recurrent non-ST-segment elevation myocardial infarctions (NSTEMI), despite no angiographic evidence of acute coronary lesions. Multimodality imaging revealed early pannus overgrowth around the THV, causing mechanical obstruction of the left main ostium. Following Heart Team discussion, she underwent successful coronary artery bypass grafting without the need for surgical THV intervention.

**Discussion:**

This case highlights a rare cause of DCO post-TAVI. Both orthotopic chimney stenting and THV explant with surgical valve replacement were deemed unsuitable in this context.

Learning points

- Early pannus formation after TAVI is a rare but significant cause of delayed coronary obstruction.
- Cardiac computed tomography is essential to identify pannus and distinguish it from thrombus or hypo-attenuated leaflet thickening (HALT).
- Multimodal imaging and Heart Team-based decision-making are key in managing complex post-TAVI cases.

## Introduction

Coronary obstruction is a rare (<1%) yet life-threatening complication of transcatheter aortic valve implantation (TAVI), associated with a 30-day mortality rate of approximately 50%.^1,2^ Most cases occur acutely, either during or shortly after the procedure. Delayed coronary obstruction (DCO), especially due to pannus formation,^3^ is exceedingly rare and poorly characterized.^4,5^

## Summary figure

**Figure:**
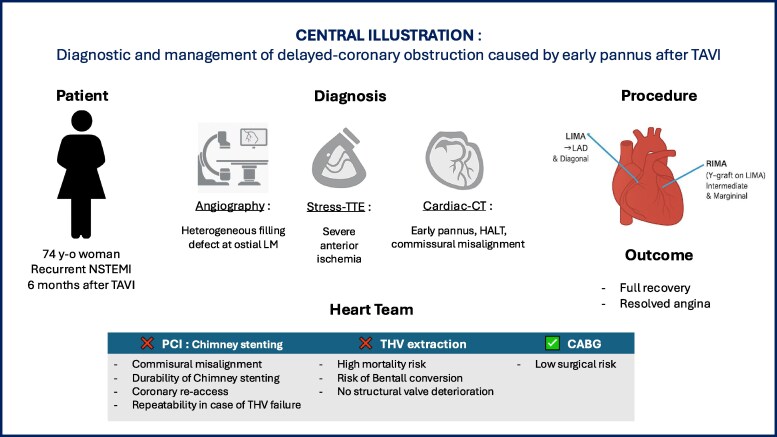
CABG: Coronary artery bypass graft; CT: computed-tomography; HALT: Hypo-Attenuated Leaflet Thickening; LAD: left anterior descending artery; LIMA: left internal mammary artery; LM: left main coronary artery; NSTEMI: non-ST-elevation myocardial infarction; PCI: percutaneous coronary intervention; RIMA: right internal mammary artery; TAVI: *trans*-aortic valve implantation; THV: transcatheter heart valve; TTE: *trans*-thoracic-echocardiography.

## Case presentation

A 74-year-old woman with no prior history of coronary artery disease was admitted to our hospital for a non-ST-segment elevation myocardial infarction (NSTEMI), occurring six months after TAVI with a 26-mm Evolut Pro+ transcatheter heart valve (THV) (Medtronic, Minneapolis).

Following the procedure, she remained asymptomatic for three months but subsequently experienced recurrent chest pain. She twice presented to the emergency department with elevated troponin levels. Repeated transthoracic echocardiography (TTE) was unremarkable. Coronary angiography with non-selective catheterization of the left main coronary artery (LM), due to self-expandable THV, showed no coronary artery stenosis.

At the time of admission, her physical examination was normal. Electrocardiogram (ECG) revealed sinus rhythm and a known left bundle branch block. High-sensitivity cardiac troponin was elevated to 4000 ng/L (normal <14 ng/L).

Her medical history included hypertension, diabetes, dyslipidaemia, peripheral artery disease, and transient ischaemic attack.

Given a history of hypertensive crises (blood pressure reaching up to 200/100 mmHg), initial episodes were attributed to type 2 myocardial infarction caused by oxygen supply-demand mismatch, possibly compounded by functional angina and modest troponin elevation. The absence of obstructive angiographic stenoses supported this hypothesis initially.

TTE showed normal left ventricular ejection fraction with known left ventricular hypertrophy, and stable prosthetic valve function (mean gradient 9 mmHg, maximum velocity 1.8 m/s) without paravalvular leak.

Repeat coronary angiography again revealed no significant acute coronary lesions to explain the clinical presentation. However, non-selective contrast injection into the LM demonstrated a heterogeneous filling defect at the ostium (see Supplementary material online, *Video S1*). The nature of the lesion—whether thrombotic, calcific, or tissue-related—could not be determined angiographically.

**Figure 1:**
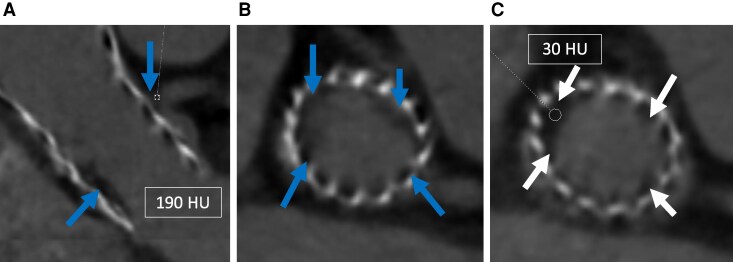
Cardiac-CT. (*A*, *B*) Circumferential pannus extending along the prosthesis up to the LM (190 HU, blue arrows). (*C*) HALT at the THV leaflet bases (30 HU, white arrows).

A multimodality imaging strategy was adopted, consistent with current ESC recommendations for complex cases of myocardial injury without angiographic culprit lesions.^6^ Cardiac computed tomography (CT) identified a circumferential soft tissue surrounding the THV, consistent with early pannus formation. Hypoattenuated leaflet thickening (HALT) was also noted at the base of the THV leaflets. The peri-valvular tissue density was 190 Hounsfield units (HU), supporting the diagnosis of pannus (*Figure 1*). Additionally, a significant commissural misalignment was observed.

Given uncertainty regarding pannus involvement, a stress TTE was performed. At 50 watts, severe inducible ischaemia occurred, with a marked drop in left ventricular ejection fraction from 55% to 20%, and new-onset anterior wall akinesia (6 out of 17 segments). These findings confirmed dynamic ischaemia in the LM territory (see Supplementary material online, *Video S2*).

We concluded that early pannus formation caused progressive mechanical LM obstruction, resulting in recurrent NSTEMIs 6 months after TAVI.

In accordance with the 2025 ESC/EACTS Guidelines for Valvular Heart Disease and given the absence of structural valve deterioration (SVD), the severity of LM obstruction and the patient’s low surgical risk (STS and EuroSCORE II <4%) supported the decision to perform coronary artery bypass grafting.^7^ She underwent successful revascularization with a left internal mammary artery graft to the left anterior descending artery and diagonal branch, and a right internal mammary artery Y-graft to the intermediate and lateral branches.

Because of HALT and high thromboembolic risk, a full-dose direct oral anticoagulant was started postoperatively.

At the 3-month follow-up, she was asymptomatic without recurrent cardiac events.

## Discussion

Pannus is a fibrotic, tissue-like overgrowth that may arise from the native aortic root or form around the frame of THV, potentially impinging on adjacent structures such as the coronary ostia. While pannus formation is a well-recognized complication following surgical aortic valve replacement (SAVR), its occurrence after TAVI remains poorly understood and underreported. Risk factors for pannus development after TAVI setting remain unclear. Jabbour *et al.* have proposed that persistent turbulent flow across the prosthesis, low-grade inflammatory responses, and valve-in-valve procedures may play a role. Interestingly, this phenomenon appears to be more frequently observed when self-expanding valves are used during the index procedure.^2^ Although not clearly linked to pannus, commissural misalignment is associated with HALT and may promote abnormal haemodynamics by inducing persistent turbulent flow across THV, conducive to pannus growth.^8^ This, in turn, could promote pannus formation, either directly through mechanical stress or indirectly via sustained low-grade inflammation.^8^

Cardiac-CT has emerged as an essential modality for post-THV failure assessment, allowing for differentiation between SVD (e.g. leaflet thickening or calcification), HALT, and pannus—typically identified by hypoattenuated tissue (with attenuation >145 HU) inside the THV frame extending up from the inflow.^7,9^ In this case, CT revealed HALT at the leaflet bases and a circumferential pannus extending along the prosthesis up to the LM. Importantly, there was no evidence of SVD. These findings align with a Japanese series,^3^ reporting pannus formation without associated SVD, albeit with limited sample size and short-term follow-up.

Here, the pannus developed as early as six months post-TAVI with a self-expandable Evolut Pro+ THV, leading to progressive and dynamic obstruction of the LM. Recurrent NSTEMI without overt coronary lesions made the diagnosis particularly challenging. As illustrated in the ESC guidelines, multimodality imaging—including cardiac-CT and stress-TTE—was essential to demonstrate both the structural abnormality and its functional significance, particularly in documenting dynamic LM ischaemia.^7^

Several management strategies were discussed within the Heart Team. Although orthotopic snorkel chimney stenting of the LM was initially considered,^4^ it was dismissed after a comprehensive evaluation of multiple factors. Significant commissural misalignment rendered the procedure technically complex and less predictable. Moreover, the lack of robust long-term data regarding the durability of chimney configurations, particularly in relatively younger patients, raised additional concerns. A recent study reported a 19% incidence of major adverse cardiovascular events at one year following this approach.^10^ Importantly, chimney stenting would also compromise the feasibility of future redo-TAVI in the event of SVD, further limiting long-term treatment options. Surgical THV explant was also ruled out. The self-expandable Evolut valve has a tall frame that extends high into the ascending aorta, increasing the risk of complex surgical conversion to a Bentall procedure and making aortic clamping particularly difficult.^11^ Indeed, THV explant is associated with high perioperative risk, with reported 30-day and 1-year mortality rates of approximately 15% and 30%, respectively.^12^ In contrast, given the absence of SVD and the presence of isolated coronary obstruction in a low surgical risk patient, a focused surgical revascularization strategy—with left and right internal mammary arteries—was considered the safest and most effective option.^7^

## Patient perspective

The patient was actively involved in all stages of care. She was fully informed and engaged in the decision-making process, and her preferences were considered during each step of management.

## Conclusions

This case highlights early pannus formation as a rare but serious aetiology of DCO, even in the absence of SVD. Cardiac CT is essential to identify pannus and distinguish it from thrombus or HALT. In line with the ESC guidelines, it underscores the importance of multimodality imaging and a multidisciplinary Heart Team approach in the management of complex post-TAVI scenarios.

## Lead author biography


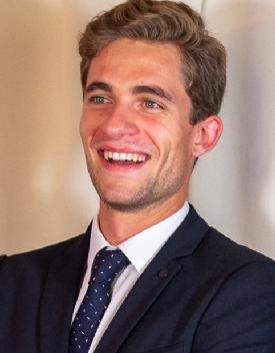


Louis Perrard, M.D., is a cardiology resident at Amiens University Hospital, France. He is involved in interventional cardiology and clinical research, with particular interest in structural heart disease. He is currently pursuing a Master’s degree in methodology and biostatistics at Université Paris-Saclay (MIRACL.AI lab, Lariboisiere Hospital, AP-HP, France) and is an active member of the French Young Cardiologists Association.

## Supplementary Material

ytag243_Supplementary_Data

## Data Availability

All data supporting the findings of this case are contained within the article and its Supplemental files. Additional de-identified patient data can be obtained from the corresponding author upon reasonable request.

## Supplementary material

**Consent:** The authors confirm that witnessed verbal consent for submission and publication of this case report including images and associated text has been obtained from the patient. This has been discussed with the editors.

## References

[1] Ojeda S, González-Manzanares R, Jiménez-Quevedo P, Piñón P, Asmarats L, Amat-Santos I, et al Coronary obstruction after transcatheter aortic valve replacement: insights from the Spanish TAVI registry. JACC Cardiovasc Interv 2023;16:1208–1217. 37225292 10.1016/j.jcin.2023.03.024

[2] Jabbour RJ, Tanaka A, Finkelstein A, Mack M, Tamburino C, Van Mieghem N, et al Delayed coronary obstruction after transcatheter aortic valve replacement. J Am Coll Cardiol 2018;71:1513–1524. 29622157 10.1016/j.jacc.2018.01.066

[3] Iwata J, Hayashida K, Kajino A, Sakata S, Ryuzaki T, Tsuruta H, et al Possible pannus formation after transcatheter aortic valve replacement with self-expandable valves. JACC Asia 2025;5:315–317. 39967224 10.1016/j.jacasi.2024.10.004 PMC11840228

[4] Akodad M, Chuang A, Ihdayhid A, Chatfield AG, Leipsic J, Cheung A, et al Early transcatheter heart valve pannus leading to coronary obstruction managed with orthotopic chimney stenting. CJC Open 2022;4:509–511. 35607485 10.1016/j.cjco.2022.01.008 PMC9123377

[5] Iwata J, Hayashida K, Shimizu H, Ieda M. Late hypo-attenuated annular narrowing 1 year after transcatheter aortic valve implantation with a navitor valve. Eur Heart J Case Rep 2024;8:ytae224. 38715623 10.1093/ehjcr/ytae224 PMC11075106

[6] Byrne RA, Rossello X, Coughlan JJ, Barbato E, Berry C, Chieffo A, et al 2023 ESC guidelines for the management of acute coronary syndromes: developed by the task force on the management of acute coronary syndromes of the European Society of Cardiology (ESC). Eur Heart J 2023;44:3720–3826. 37622654 10.1093/eurheartj/ehad191

[7] Praz F, Borger MA, Lanz J, Marin-Cuartas M, Abreu A, Adamo M, et al 2025 ESC/EACTS guidelines for the management of valvular heart disease. Eur Heart J 2025;46:4635–4736. 40878295 10.1093/eurheartj/ehaf194

[8] Jung S, Ammon F, Smolka S, Moshage M, Marwan M, Achenbach S. Commissural misalignment independently predicts leaflet thrombosis after transcatheter aortic valve implantation. Clin Res Cardiol Off J Ger Card Soc 2024;113:29–37. 10.1007/s00392-023-02192-6 PMC10808532 37022472

[9] Khalique OK, Zaid S, Tang GHL, Abdel-Wahab M, Akodad M, Bapat M, et al Best practices for imaging of transcatheter valve failure: an update from the heart valve collaboratory. J Am Coll Cardiol 2025;85:1042–1055. 40074470 10.1016/j.jacc.2024.12.017

[10] Mangieri A, Richter I, Gitto M, Abdelhafez A, Bedogni F, Lanz J, et al Chimney stenting vs BASILICA for prevention of acute coronary obstruction during transcatheter aortic valve replacement. JACC Cardiovasc Interv 2024;17:742–752. 38538170 10.1016/j.jcin.2024.01.007

[11] Tang GHL, Zaid S, Kleiman NS, Goel SS, Fukuhara S, Marin-Cuartas M, et al Explant vs redo-TAVR after transcatheter valve failure: mid-term outcomes from the EXPLANTORREDO-TAVR international registry. JACC Cardiovasc Interv 2023;16:927–941. 37100556 10.1016/j.jcin.2023.01.376

[12] Belluschi I, Buzzatti N, Romano V, De Backer O, Søndergaard L, Karady J, et al Surgical feasibility of ascending aorta manipulation after transcatheter aortic valve implantation: a computed tomography theoretical analysis. EuroIntervention J Eur Collab Work Group Interv Cardiol Eur Soc Cardiol 2021;16:e1533–e1540. 10.4244/EIJ-D-19-00991 PMC9724966 32364502

